# Adolescent Bullying: Bridging the Past and Present

**DOI:** 10.1192/j.eurpsy.2025.444

**Published:** 2025-08-26

**Authors:** N. E. Ayadi, S. Bourgou, F. Charfi, M. al’Absi, A. Ghenimi

**Affiliations:** 1 child and adolescent psychiatry, mongi slim hospital, La Marsa Tunis, Tunisia; 2 University of Minnesota Medical School, University of Minnesota Medical School, minnesota, United States; 3 child and adolescent psychiatry department, Mongi Slim university Hospital, Tunis, Tunisia

## Abstract

**Introduction:**

Bullying is a persistent aggressive behavior characterized by a power imbalance and driven by the intent to cause harm. Examining the connection between past experiences of victimization and current bullying behaviors is essential for a comprehensive understanding of this phenomenon..

**Objectives:**

This study aims to assess the prevalence of bullying and cyberbullying among Tunisian adolescents and to explore the relationship between current bullying behaviors and a history of childhood victimization.

**Methods:**

This cross-sectional study was conducted via a survey among adolescents attending middle and high schools during the 2023-2024 school year. Participants provided written consent and completed a demographic information sheet, the Adolescents and Peer Relations Instrument (APRI), and the Adverse Childhood Experience-International Questionnaire (ACE-IQ).

**Results:**

The study population consisted of 1,005 adolescents, with a sex ratio of 0.73 and a mean age of 14.62 years. We find that 92.1% of adolescents experienced traditional bullying within the year, with 54.9% exposed to school bullying more than once a month. The most prevalent form was verbal bullying (88%), followed by relational bullying (77.3%) and physical bullying (73.9%). Among the participants, 38.5% had no history of past bullying but were currently victimized, 53.7% had experienced bullying in the past and continued to be victimized, 1.3% had been victimized in the past but no longer experienced bullying, and 6.5% had never been bullied, either in the past or present.

We identified a significant association between peer violence and bullying victimization, with a p-value <0.001 and an odds ratio (OR) of 4.11 (95% CI: 2.5-6.66). There was also a significant correlation between peer violence and the APRI scale (p < 0.001). Further analysis showed a strong correlation between peer violence and verbal bullying victimization (p < 0.001, OR = 3.75 [2.53-5.56]), relational bullying victimization (p < 0.001, OR = 2.8 [2.1-3.8]), and physical bullying victimization (p < 0.001, OR = 2.64 [1.97-3.53]). The risk of being victimized by all three forms of bullying was tripled (p < 0.001, OR = 2.75 [2.1-3.6]).

**Conclusions:**

This study highlights the alarmingly high prevalence of bullying among Tunisian adolescents and emphasizes the significant link between past victimization and current bullying experiences. These findings underscore the need for targeted interventions that address the underlying trauma from past experiences to promote positive youth development.

**Disclosure of Interest:**

None Declared

